# Derivation and external validation of a risk prediction algorithm to estimate future risk of cardiovascular death among patients with type 2 diabetes and incident diabetic nephropathy: prospective cohort study

**DOI:** 10.1136/bmjdrc-2019-000735

**Published:** 2019-11-13

**Authors:** Dahai Yu, Jin Shang, Yamei Cai, Zheng Wang, Xiaoxue Zhang, Bin Zhao, Zhanzheng Zhao, David Simmons

**Affiliations:** 1 Department of Nephrology, the First Affiliated Hospital, Zhengzhou University, Zhengzhou, China; 2 Primary Care Centre Versus Arthritis, Research Institute for Primary Care & Health Sciences, Keele University, Keele, UK; 3 The Second Division of Internal Medicine, Kejing Community Health Centre, Jiyuan, China; 4 Western Sydney University, Campbelltown, Sydney, New South Wales, Australia

**Keywords:** cardiovascular mortality, prediction, statistical, prognostic models

## Abstract

**Objective:**

To derive, and externally validate, a risk score for cardiovascular death among patients with type 2 diabetes and newly diagnosed diabetic nephropathy (DN).

**Research design and methods:**

Two independent prospective cohorts with type 2 diabetes were used to develop and externally validate the risk score. The derivation cohort comprised 2282 patients with an incident, clinical diagnosis of DN. The validation cohort includes 950 patients with incident, biopsy-proven diagnosis of DN. The outcome was cardiovascular death within 2 years of the diagnosis of DN. Logistic regression was applied to derive the risk score for cardiovascular death from the derivation cohort, which was externally validated in the validation cohort. The score was also estimated by applying the United Kingdom Prospective Diabetes Study (UKPDS) risk score in the external validation cohort.

**Results:**

The 2-year cardiovascular mortality was 12.05% and 11.79% in the derivation cohort and validation cohort, respectively. Traditional predictors including age, gender, body mass index, blood pressures, glucose, lipid profiles alongside novel laboratory test items covering five test panels (liver function, serum electrolytes, thyroid function, blood coagulation and blood count) were included in the final model.

C-statistics was 0.736 (95% CI 0.731 to 0.740) and 0.747 (95% CI 0.737 to 0.756) in the derivation cohort and validation cohort, respectively. The calibration slope was 0.993 (95% CI 0.974 to 1.013) and 1.000 (95% CI 0.981 to 1.020) in the derivation cohort and validation cohort, respectively.

The UKPDS risk score substantially underestimated cardiovascular mortality.

**Conclusions:**

A new risk score based on routine clinical measurements that quantified individual risk of cardiovascular death was developed and externally validated. Compared with the UKPDS risk score, which underestimated the cardiovascular disease risk, the new score is a more specific tool for patients with type 2 diabetes and DN. The score could work as a tool to identify individuals at the highest risk of cardiovascular death among those with DN.

**Significance of this study**

**What is already known about this subject?**

- Cardiovascular disease has been found to be the primary cause of death in people with diabetic nephropathy (DN).
- No risk scores had been developed and externally validated to predict short-term cardiovascular mortality in cohorts for patients with DN and type 2 diabetes.

**What are the new findings?**

- Using two independent prospective cohorts, a new risk score to predict cardiovascular death within 2 years since the diagnosis of DN was developed and externally validated with good discrimination and calibration.
- Unlike the new score, the UKPDS score substantially underestimated cardiovascular mortality.

**How might these results change the focus of research or clinical practice?**

- The score is derived based on routine clinical measurements that are commonly available for patients with DN either in an outpatient setting or in the inpatient setting.
- The score could work as a screening tool to identify individuals at the highest risk of cardiovascular death among those with DN.

## Background

Diabetic nephropathy (DN) is one of the most significant complications of diabetes mellitus and the most frequent cause of end-stage renal disease.^1^ Cardiovascular complications, induced by accelerating arteriosclerosis, comprise nearly 50% of all comorbidity and mortality in patients with type 2 diabetes and those with renal insufficiency caused by diabetes have a yet greater risk of cardiovascular complications.^2^ To reduce future cardiovascular mortality risk among patients with DN, risk algorithms predicting future individual absolute risk of cardiovascular mortality are required to help clinicians and patients to assess the management status and develop personalised care strategies.^3^ In previous studies, a number of prognostic factors have been identified including microalbuminuria,^4^ hypothyroidism,^5^ osteopontin^6^ and the metabolic syndrome.^7^ However, few studies have calculated risk algorithms to identify those with DN, and those who are at particularly high risk of cardiovascular mortality, especially short-term cardiovascular mortality.

The aim of the present study was to develop and validate a multivariable risk algorithm to predict cardiovascular deaths within 2 years of diagnosis of type 2 DN among patients with type 2 diabetes.

## Methods

### Data source and study population

We used two independent prospective cohorts from the First Affiliated Hospital of Zhengzhou University, Henan, China: one (derivation) based on the electronic health record data from outpatient and inpatient registries to develop our cardiovascular mortality risk score and another (validation) based on biopsy registry cohort data for external validation. The diagnosis of type 2 diabetes was based on the American Diabetes Association criteria.^8^

## Derivation cohort

A total 2282 patients in the derivation cohort were enrolled through the outpatient and inpatient departments (except Department of Nephrology) in the First Affiliated Hospital of Zhengzhou University. This is the largest hospital in China and provides both primary and secondary care to Henan province residents. To develop a risk score that could potentially be applied to the general DN population, all patients with incident (based on patients’ previous medical records) DN clinically diagnosed in the hospital between 1 January 2015 and 31 December 2016 were enrolled as the derivation cohort. Each patient’s clinical diagnosis date was recorded as the enrol date (baseline examination date). DN was defined as the presence of nephropathy in patients with type 2 diabetes and albuminuria >300 mg/g creatinine; or patients with diabetes and albuminuria >30–300 mg/g creatinine and an estimated glomerular filtration rate (eGFR) <60 mL/min/1.73 m^2^.^9^

### External validation cohort

The study included 950 patients with type 2 diabetes with biopsy-proven DN newly diagnosed between 1 January 2015 and 31 December 2016. Each patient’s biopsy-proven diagnosis date was recorded as the enroll date (baseline examination date). Patients were either outpatients or inpatients under the Department of Nephrology, the First Affiliated Hospital of Zhengzhou University. The diagnosis of DN was made based on histological characteristics, including glomerular hypertrophy, thickened capillary basement membranes, diffuse mesangial expansion (sclerosis), nodular mesangial sclerosis, exudative lesions such as capsular drop or fibrin cap, mesangiolysis, mescapillary microaneurysm or hyalinosis of afferent and efferent arterioles, using appropriate standards for renal biopsy including light microscopy, electron microscopy and immunofluorescence examination.^10^ Patients with other glomerular disease concomitant with DN were excluded from this study. Renal biopsy was performed for precise diagnosis of renal lesions with the consent of each patient. The biopsy results were reviewed by three clinicians and the diagnosis was made only based on the agreement of more than two clinicians.

### Defining cardiovascular death

Every patient included in both the derivation cohort and the validation cohort were followed up for 2 years following the baseline examination, for example, the follow-up time would be 5 January 2017 if the baseline examination was 6 January 2015. We defined the primary outcome as death with cardiovascular disease as the primary diagnosis over the 2-year follow-up. An outcome assessment committee at the First Affiliated Hospital of Zhengzhou University reviewed medical history and death certificates and determined the final underlying cause of death. Two clinicians in the committee independently verified the diagnosis, and discrepancies were adjudicated by discussion involving additional committee members. All clinicians in the committee were unaware of patient’s baseline status.

### Candidate predictors, missing data and power calculations

Two demographic characteristics (age and gender), three clinical measurements (body mass index (BMI), systolic blood pressure (SBP) and diastolic blood pressure (DBP)) and six glucose and lipid profiles measurements (fasting glucose, hemoglobin A1c (HbA1c), total cholesterol, high-density lipoprotein (HDL) cholesterol, low-density lipoprotein (LDL) cholesterol, triglyceride) and 62 laboratory test items (covering full blood count, liver function and blood coagulation tests, serum electrolytes and renal function) routinely measured among patients with incident type 2 DN in both outpatient clinics and inpatient departments were initially identified. To minimize overfitting of the model and maintain the proper statistical power, all 62 laboratory test items were independently reviewed and combined into 16 integrated predictors (online supplementary table 1) based on the clinical utilization by two clinicians at the First Affiliated Hospital of Zhengzhou University. Discrepancies were adjudicated by discussion involving an additional clinician. In total, 27 potential predictors were selected for the subsequent model development.

10.1136/bmjdrc-2019-000735.supp1 Supplementary data

Our derivation cohort had missing information on BMI (26.2%), SBP (27.4%), DBP (27.2%), fasting glucose (2.1%), hematocrit (3.6%), mean corpuscular hemoglobin (3.6%), mean platelet volume (3.6%), monocyte (3.6%), red blood cell distribution width (3.6%), magnesium (12.1%), sodium (12.1%), chlorine (12.1%), activated partial thromboplastin time (22.8%), D-dimer (12.8%), thrombin time (22.8%), fibrinogen (22.8%) and fibrinogen degradation products (22.8%). We used multiple imputation to replace missing values by using a chained equation approach based on all candidate predictors and outcomes. We created 27 imputed datasets for missing variables that were then combined across all datasets by using Rubin’s rule to obtain final model estimates.^11^

Using the same method, we also imputed values for patients with missing information on BMI (16.2%), SBP (17.4%), DBP (22.2%), fasting glucose (22.1%), hematocrit (2.6%), mean corpuscular hemoglobin (2.6%), mean platelet volume (2.6%), monocyte (2.6%), red blood cell distribution width (12.7%), magnesium (12.5%), sodium (2.1%), chlorine (12.7%), activated partial thromboplastin time (16.7%), D-dimer (16.7%), thrombin time (16.7%), fibrinogen (22.7%) and fibrinogen degradation products (16.7%) in the external validation cohort.

On the basis of 275 cardiovascular deaths and all 27 potential predictors before backward elimination in our derivation cohort, we had an effective sample size of 10.2 cardiovascular deaths per parameter, above the minimum requirement suggested by Peduzzi *et al*.^12^

### Ethical approval

Written informed consent was obtained from all participants before inclusion.

### Statistical analysis for model derivation and external validation

We treated occurrence of cardiovascular deaths within 2 years of the beginning of follow-up, as binary outcome measures. For each of the 27 potential predictors (covering 73 items as described above), we used a univariable logistic regression model to calculate the unadjusted ORs. For derivation of the risk prediction model, we initially included all candidate predictors in a multivariable logistic regression model. We tried to use both fractional polynomial terms and binary terms (using medians of measurements in the derivation cohort as cut-offs) of continuous predictors (age, BMI, SBP, DBP, HbA1c, glucose, total cholesterol, HDL, LDL and triglyceride). The term with best model fit statistics (minimum Bayesian information criterion (BIC)) in the full model was used as the term for above-mentioned continuous predictors.

Through backward elimination, we excluded five predictors (covering 19 items) from the multivariate model as they were not statistically significant (p>0.1 based on change in log likelihood).^13^ After elimination, we reinserted the excluded predictors into the final model to further check whether it became statistically significant. We also rechecked fractional polynomial terms at this stage and re-estimated them where necessary. Finally, 27 parameters (binary or polynomial terms) from 22 predictors remained in the final model.

We formed risk equations for predicting the log odds of cardiovascular death by using the estimated regression coefficients multiplied by the corresponding predictors included in our model together with the intercepts. This process ultimately led to equations for the predicted risk=1/(1+e^−riskscore^), where the ‘risk score’ is the predicted log odds of cardiovascular death from the developed model.

To facilitate model utilization in clinical practice, the logistic regression equations were transformed into prognostic score charts. The coefficients in the logistic regression equation were multiplied by 12.5 and rounded to the nearest integer to obtain the prognostic score per predictor. Multiplication by 12.5 was chosen to place the majority of the coefficients close to an integer, thereby minimizing the effects of rounding. The sum of all prognostic scores reflects patients’ probability of cardiovascular deaths.^14^

We assessed the performance of the models in terms of the C-statistic and calibration slope (where 1.00 is ideal). The C-statistic represents the probability that for any randomly selected pair of people with DN with and without outcomes, the patient with the outcome had a higher predicted risk.^15^ A value of o.50 indicated no discrimination and 1.00 represents perfect discrimination. We then undertook internal validation to correct measures of predictive performance for optimism (overfitting) by bootstrapping 100 samples of the derivation data. We repeated the model derivation process in each bootstrap sample to produce a model, applied the model to the same bootstrap sample to quantify apparent performance and applied the model to the original dataset to test model performance (calibration slope and C-statistic) and optimism (difference in the test performance and apparent performance). We then estimated the overall optimism across all models.

We applied our risk prediction model to each patient with DN in the external validation cohort on the basis of the presence of one or more predictors. We examined the performance of this final model both in the derivation dataset and then in the external validation dataset in terms of discrimination by calculating the C-statistic. We examined calibration by plotting agreement between predicted and observed risks across tenth of the predicted risks.

We also applied the United Kingdom Prospective Diabetes Study (UKPDS) risk score^16^ in the external validation cohort to estimate the 2-year risk of cardiovascular disease (CVD) in terms of model calibration, to test whether this general risk score for patients with type 2 diabetes would still be suitable for patients with type 2 diabetes and DN. As the smoking information was not accessible in this study, estimates were made assuming both the lowest (all non-smoking) and highest (all smoking) scenarios.

We used Stata V.15.0 for all statistical analyses. This study was conducted and reported in line with the Transparent Reporting of a multivariate prediction model for Individual Prediction Diagnosis guidelines.^17^

## Results

### Study participants

In our derivation cohort, we analyzed information on 2282 patients with DN with 275 cardiovascular deaths within 2 years. Our validation cohort had information on 950 patients with DN with 112 cardiovascular deaths. Table 1 summarizes the basic characteristics and potential predictors of the study population. For general characteristics, patients in the derivation cohort had higher age, higher proportion of males and higher DBP and SBP compared with those in the validation cohort. Test items within the thyroid function test panel, blood coagulation test panel, full blood count test panel and lipid profile test panel were similar between the derivation cohort and the validation cohort. Most test items in the liver function test panel were generally higher among patients in the derivation cohort compared with those in the validation cohort, except for pre-albumin and alkaline phosphate which were lower in the derivation cohort; and cholinesterase, albumin and globulin which were similar between the derivation cohort and the validation cohort. Most test items in the renal function test panel were generally lower in the derivation cohort compared with those in the derivation cohort, except for eGFR which was higher in the derivation cohort, and pH of the urine sample and urine-specific gravity which were similar between the derivation cohort and the validation cohort.

**Table 1:** Baseline characteristics of study cohorts

Candidate predictor	Test domain	Derivation cohort n=2282	Validation cohort n=950
Cardiovascular deaths, n (%)	Outcome	275 (12.05%)	112 (11.79%)
Age, years	General	59.00 (50.00 to 67.00)	56.50 (48.00 to 62.00)
Male gender, n (%)	General	1350 (59.15%)	535 (56.32%)
Systolic blood pressure, mm Hg	General	150.00 (135.00 to 167.00)	156.00 (140.00 to 171.00)
Diastolic blood pressure, mm Hg	General	89.00 (80.00 to 97.00)	82.00 (52.00 to 96.00)
Body mass index, kg/m^2^	General	26.00 (20.35 to 32.15)	26.00 (19.39 to 28.48)
Direct bilirubin, µmol/L	Liver function	3.40 (2.30 to 5.10)	2.35 (1.50 to 3.10)
Cholinesterase, U/L	Liver function	6.85 (4.70 to 9.25)	6.77 (3.40 to 9.13)
Pre-albumin, mg/dL	Liver function	234.04 (185.33 to 282.00)	246.27 (193.90 to 284.20)
Total bile acid, µmol/L	Liver function	3.90 (1.85 to 7.68)	3.30 (1.90 to 7.62)
Albumin, g/L	Liver function	39.60 (32.20 to 60.83)	36.60 (25.40 to 43.26)
Alkaline phosphatase, IU/L	Liver function	74.00 (60.00 to 93.00)	81.75 (65.13 to 94.75)
Alanine transaminase, U/L	Liver function	16.00 (11.00 to 24.00)	12.00 (9.00 to 21.75)
Gamma-glutamyl transferase, U/L	Liver function	23.00 (15.00 to 39.50)	18.00 (12.25 to 34.88)
Globulin, g/L	Liver function	25.48 (22.40 to 29.10)	25.25 (21.11 to 30.88)
Indirect bilirubin, µmol/L	Liver function	3.70 (2.40 to 5.70)	2.40 (1.69 to 4.10)
Total bilirubin, µmol/L	Liver function	7.20 (4.90 to 10.75)	4.83 (3.45 to 7.13)
Total protein, g/dL	Liver function	63.40 (57.50 to 68.10)	61.64 (53.20 to 65.98)
Aspartate transaminase, U/L	Liver function	17.00 (13.00 to 22.67)	14.42 (11.00 to 18.00)
24 hours total urine protein, g	Renal function	2.80 (0.09 to 3.57)	3.38 (1.49 to 5.67)
24 hours total urine amount, L	Renal function	2.00 (1.00 to 3.50)	3.26 (1.50 to 33.87)
Urine microalbumin, mg/L/24 hours	Renal function	149.07 (18.00 to 940.00)	179.27 (35.00 to 958.00)
pH of urine sample	Renal function	6.00 (5.75 to 6.45)	6.00 (5.70 to 6.50)
Urine-specific gravity	Renal function	1.01 (1.00 to 1.02)	1.01 (1.00 to 1.02)
Urine total protein, mg/24 hours	Renal function	421.10 (43.00 to 2078.86)	467.30 (255.25 to 3225.92)
Creatinine, μmoI/L	Renal function	86.00 (62.00 to 200.00)	117.25 (68.50 to 417.25)
Urine acid, µmol/L	Renal function	303.00 (241.00 to 378.00)	326.00 (265.00 to 383.00)
Estimated glomerular filtration rate, mL/min/1.73 m^2^	Renal function	35.79 (14.35 to 53.42)	32.13 (12.63 to 46.38)
Urea, mmol/L	Renal function	7.38 (5.35 to 12.62)	9.73 (5.59 to 18.37)
Magnesium, mmol/L	Serum electrolytes	0.93 (0.86 to 1.01)	0.96 (0.87 to 1.04)
HCO_3_, mEq/L	Serum electrolytes	23.60 (21.30 to 25.90)	22.50 (19.35 to 25.70)
Calcium, mmol/L	Serum electrolytes	2.23 (2.11 to 2.31)	2.13 (2.05 to 2.27)
Chlorine, mmol/L	Serum electrolytes	102.30 (99.80 to 105.20)	102.00 (99.00 to 107.33)
Potassium, mmol/L	Serum electrolytes	4.36 (4.01 to 4.70)	4.53 (4.29 to 4.90)
Sodium, mmol/L	Serum electrolytes	141.00 (138.50 to 143.10)	140.33 (137.00 to 143.00)
Phosphorus, mmol/L	Serum electrolytes	1.23 (1.07 to 1.40)	1.33 (1.16 to 1.47)
Free triiodothyronine, pmol/L	Thyroid function	4.24 (3.63 to 4.88)	4.13 (3.36 to 4.50)
Free thyroxine, pmol/L	Thyroid function	11.93 (10.23 to 13.90)	11.43 (9.25 to 14.39)
Thyroid-stimulating hormone, µIU/mL	Thyroid function	2.04 (1.12 to 3.86)	2.83 (1.17 to 4.96)
D-dimer, µg/mL	Blood coagulation	0.15 (0.06 to 0.45)	0.17 (0.10 to 0.64)
Prothrombin time	Blood coagulation	9.80 (9.10 to 10.70)	9.70 (8.90 to 10.60)
Prothrombin time activity	Blood coagulation	128.00 (110.00 to 147.15)	126.68 (112.00 to 151.00)
Activated partial thromboplastin time, s	Blood coagulation	33.56 (30.10 to 37.00)	32.40 (29.80 to 35.60)
Fibrinogen, g/L	Blood coagulation	3.19 (2.61 to 3.96)	3.22 (2.84 to 3.87)
Thrombin time, s	Blood coagulation	14.80 (13.60 to 16.50)	15.65 (13.80 to 17.30)
International normalized ratio	Blood coagulation	0.86 (0.81 to 0.94)	0.87 (0.80 to 0.93)
Basophil, 10^9^/L	Blood count	0.03 (0.02 to 0.04)	0.03 (0.02 to 0.04)
Basophil, %	Blood count	0.50 (0.40 to 0.65)	0.53 (0.40 to 0.60)
Eosinophil granulocyte, 10^9^/L	Blood count	0.13 (0.08 to 0.20)	0.15 (0.09 to 0.24)
Eosinophil granulocyte, %	Blood count	2.00 (1.20 to 3.00)	2.20 (1.08 to 3.90)
Hematocrit, %	Blood count	0.37 (0.31 to 0.44)	0.31 (0.28 to 0.39)
Hemoglobin, g/L	Blood count	117.00 (96.00 to 135.00)	98.33 (89.13 to 123.96)
Lymphocyte, 10^9^/L	Blood count	1.70 (1.25 to 2.10)	1.39 (1.21 to 2.13)
Lymphocyte, %	Blood count	26.10 (19.38 to 33.10)	25.80 (17.50 to 32.10)
Mean corpuscular hemoglobin, pg	Blood count	29.90 (28.90 to 31.00)	29.65 (27.40 to 30.80)
Mean corpuscular hemoglobin concentration, g/L	Blood count	329.00 (323.00 to 334.00)	324.25 (320.25 to 332.88)
Mean corpuscular volume, fL	Blood count	91.00 (87.80 to 93.80)	90.00 (84.80 to 93.20)
Monocytes, 10^9^/L	Blood count	0.50 (0.40 to 0.63)	0.43 (0.36 to 0.57)
Monocytes, %	Blood count	7.50 (6.33 to 8.90)	7.65 (6.00 to 8.68)
Mean platelet volume, fL	Blood count	8.50 (7.80 to 9.30)	8.35 (7.54 to 8.98)
Neutrophil, 10^9^/L	Blood count	4.20 (3.20 to 5.50)	4.07 (3.30 to 4.93)
Neutrophil, %	Blood count	63.00 (55.40 to 70.03)	64.83 (56.50 to 70.03)
Plateletcrit, %	Blood count	0.17 (0.14 to 0.21)	0.18 (0.14 to 0.22)
Platelet distribution width, %	Blood count	16.75 (16.40 to 17.15)	16.93 (16.50 to 17.39)
Platelets, 10^9^/L	Blood count	196.00 (160.00 to 239.00)	187.00 (157.50 to 247.00)
Red blood cell, 10^12^/L	Blood count	4.29 (3.24 to 6.79)	3.57 (3.12 to 6.35)
Red blood cell distribution width, %	Blood count	13.60 (13.00 to 14.49)	13.83 (13.06 to 14.55)
White blood cell count, 10^9/^L	Blood count	6.55 (4.80 to 10.00)	6.38 (5.05 to 11.18)
Glucose, mmol/L	Blood glucose/lipids	7.66 (5.50 to 10.63)	6.80 (5.14 to 10.12)
High-density lipoprotein cholesterol, mmol/L	Blood glucose/lipids	1.05 (0.86 to 1.30)	0.95 (0.81 to 1.34)
Hemoglobin A1c, mmol/mol	Blood glucose/lipids	62.80 (49.70 to 80.30)	58.50 (53.0 to 84.70)
Low-density lipoprotein cholesterol, mmol/L	Blood glucose/lipids	2.62 (1.99 to 3.35)	2.97 (2.10 to 3.66)
Total cholesterol, mmol/L	Blood glucose/lipids	4.28 (3.54 to 5.09)	4.70 (3.74 to 5.64)
Triglyceride, mmol/L	Blood glucose/lipids	1.43 (1.02 to 2.19)	1.70 (1.00 to 2.28)

### Model derivation, performance measure and validation

**Figure 1:**
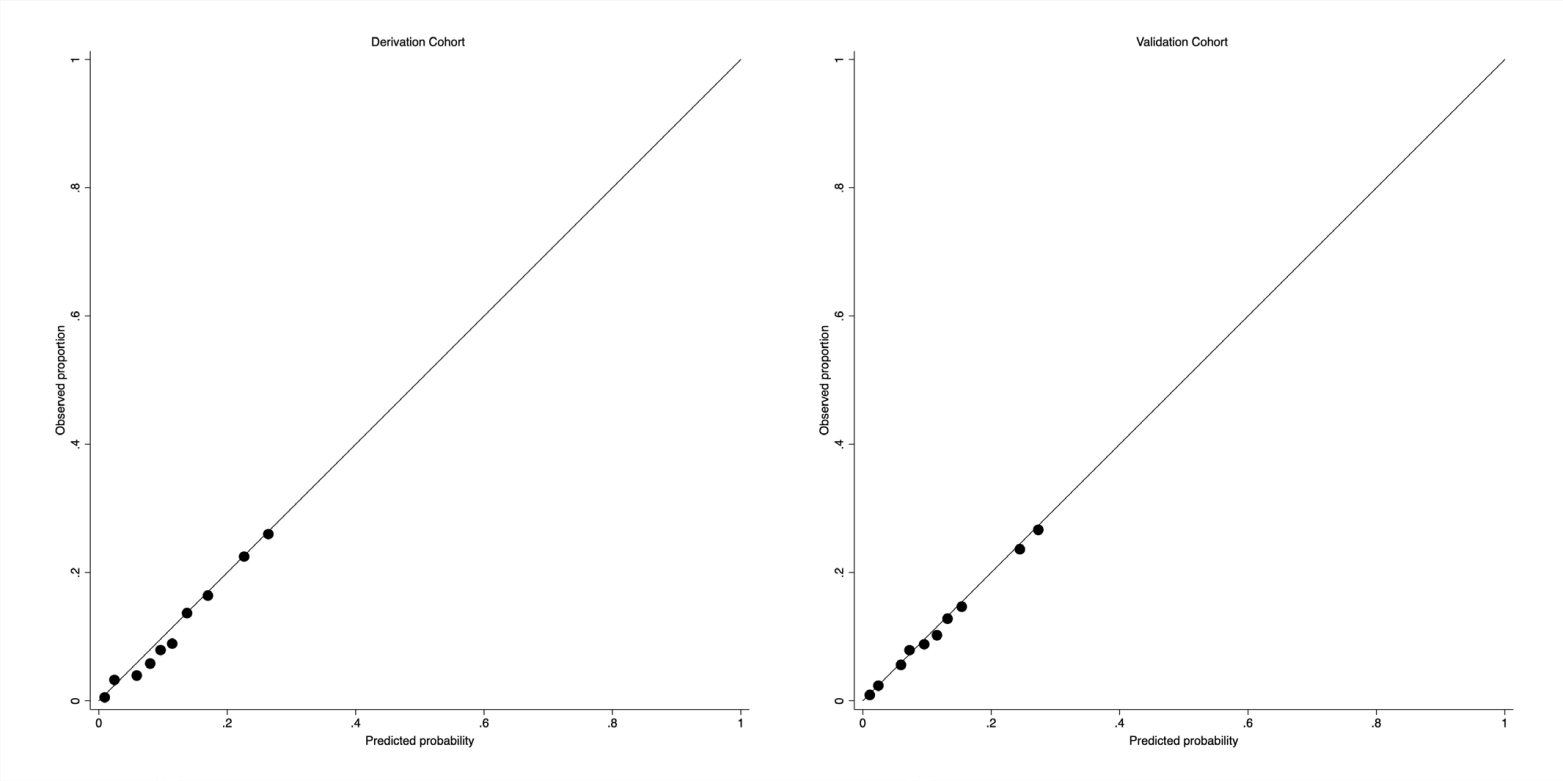
Assessing calibration in the derivation cohort (left) and the validation cohort (right) for cardiovascular death.

In the derivation dataset, the absolute risk of cardiovascular death within 2 years was 12.05%. Of the 27 candidate predictors (online supplementary table 1), 22 predictors (27 parameters) were statistically significantly associated with cardiovascular death in the final multivariate model (table 2). Table 2 shows the apparent and internal validation performance statistics of the risk prediction model. After adjustment for optimism, the final risk prediction model was able to discriminate patients with DN with and without cardiovascular death with a C-statistic of 0.736 (95% CI 0.731 to 0.740). The agreement between the observed and predicted proportion of cardiovascular hospitalization and re-hospitalization showed good apparent calibration (figure 1, left). The optimism-adjusted calibration slope was 0.993 (95% CI 0.974 to 1.013) for cardiovascular death (table 3).

**Table 2:** Final multivariate analysis for cardiovascular death risk among people with type 2 diabetes and a new clinical diagnosis of DN in derivation cohort

Parameters	Coefficients	95% CI
Gender: male vs female	0.157086	0.113221 to 0.200952
(Age/10)^3^	0.000782	−0.002189 to 0.003752
(Age/10)^3^×ln(age/10)	0.000499	−0.000851 to 0.001849
(Body mass index/10)^3^	1.057959	0.938973 to 1.176945
(Body mass index/10)^3^×ln(body mass index/10)	−0.831356	−0.923908 to−0.738804
Systolic blood pressure ≥150 mm Hg	0.132676	0.087332 to 0.178021
Diastolic blood pressure ≥90 mm Hg	0.022270	−0.022518 to 0.067057
(Fasting glucose/10)^2^	0.562659	0.480369 to 0.644950
(Fasting glucose/10)^3^	−0.207944	−0.246155 to −0.169732
(e^(high-density lipoprotein cholesterol)/10^)^−0.5^	−5.667237	−6.281152 to −5.053321
(e^(high-density lipoprotein cholesterol)/10^)^−0.5^×ln(e^(high-density lipoprotein cholesterol)/10^)	−1.998909	−2.190463 to −1.807355
{e^[log10(Triglyceride)]^}^−0.5^	0.350930	−0.475933 to 1.177792
{e^[log10(Triglyceride)]^}^−0.5^×ln{e^[log10(Triglyceride)]^}	−0.308807	−0.692824 to 0.075210
Low-density lipoprotein cholesterol ≥2.60	0.322825	0.278944 to 0.366706
International normalized ratio ≥0.86 **or** D-dimer ≥0.15 **or** fibrinogen ≥3.19 **or** thrombin time ≥14.8	1.929119	1.779267 to 2.078971
Prothrombin time activity ≥128 **or** activated partial thromboplastin time ≥33.56 **or** prothrombin time ≥9.8	1.005904	0.852349 to 1.159459
Thyroid-stimulating hormone ≥2.04 **or** free thyroxine ≥11.93 **or** free triiodothyronine ≥4.24	−0.176353	−0.234195 to −0.118511
Magnesium ≥0.93 **or** phosphorus ≥1.23 **or** potassium ≥4.36 **or** sodium ≥141	0.138010	0.061919 to 0.214101
HCO_3_ ≥23.6 **or** chlorine ≥102.3 **or** calcium ≥2.23	−0.545899	−0.601485 to −0.490313
Total bilirubin ≥7.2 **or** total protein ≥63.4	−0.741628	−0.790879 to −0.692376
Cholinesterase ≥6.85 **or** alanine transaminase ≥16 **or** gamma-glutamyl transtransferase ≥23 **or** alkaline phosphatase ≥74	−0.278849	−0.342571 to −0.215127
Direct bilirubin ≥3.4 **or** globulin ≥25.48 **or** indirect bilirubin ≥3.7	0.385626	0.324596 to 0.446655
Urine-specific gravity ≥1.01 **or** 24 hours total urine protein ≥0.86 **or** urea ≥7.38	1.789318	1.605494 to 1.973141
Basophil ≥0.03×10^9^/L **or** eosinophil granulocyte ≥0.13×10^9^/L **or** mean corpuscular hemoglobin ≥29.9 **or** platelet distribution width ≥16.75 **or** plateletcrit ≥0.169% **or** monocytes ≥7.5%	−1.364007	−1.552228 to −1.175786
Lymphocyte ≥26.1% **or** neutrophil ≥63% **or** hemoglobin ≥117	−0.476919	−0.588630 to −0.365208
Hematocrit ≥0.37 **or** red blood cell distribution width ≥13.6% **or** neutrophil ≥4.2×10^9^/L **or** mean corpuscular hemoglobin concentration ≥329	−0.228664	−0.359169 to −0.098159
Lymphocyte ≥1.7×10^9^/L **or** mean corpuscular volume ≥91 **or** monocytes×10^9^/L≥0.5	0.411002	0.346310 to 0.475694
Constant	−4.073395	−5.295054 to −2.851736

DN, diabetic nephropathy.

**Table 3:** Model diagnostics (with 95% CI)

Measure	Development				External validation
	Apparent performance	Test performance	Average optimism	Optimism corrected	
C-statistic	0.738 (0.733 to 0.742)	0.736 (0.726 to 0.746)	+0.002	0.736 (0.731 to 0.740)	0.747 (0.737 to 0.756)
Calibration slope	1.000 (0.981 to 1.019)	0.993 (0.990 to 0.997)	+0.007	0.993 (0.974 to 1.013)	1.000 (0.981 to 1.020)

### External validation

In the external validation cohort, the absolute risks for cardiovascular death was 11.79%. Applying our final risk prediction model to the independent population gave a C-statistic of 0.747 (95% CI 0.737 to 0.756) for cardiovascular death and good calibration (figure 1, right), with the calibration slope 1.000 (95% CI 0.981 to 1.020) for cardiovascular deaths.

**Figure 2:**
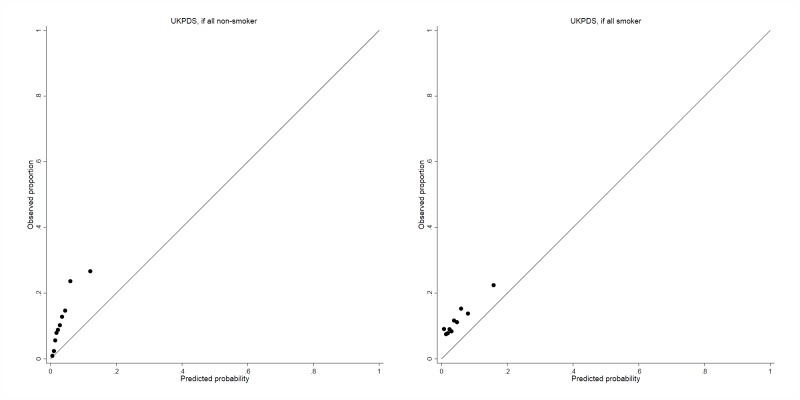
Assessing calibration in the validation cohort for cardiovascular death by applying the United Kingdom Prospective Diabetes Study (UKPDS) risk score.

A substantially underestimated cardiovascular risk was observed in the external validation cohort when applying the UKPDS risk score for 2-year risk both with (highest risk) (figure 2, right) and without (lowest risk) (figure 2, left) smoking information.

### Clinical examples

**Figure 3:**
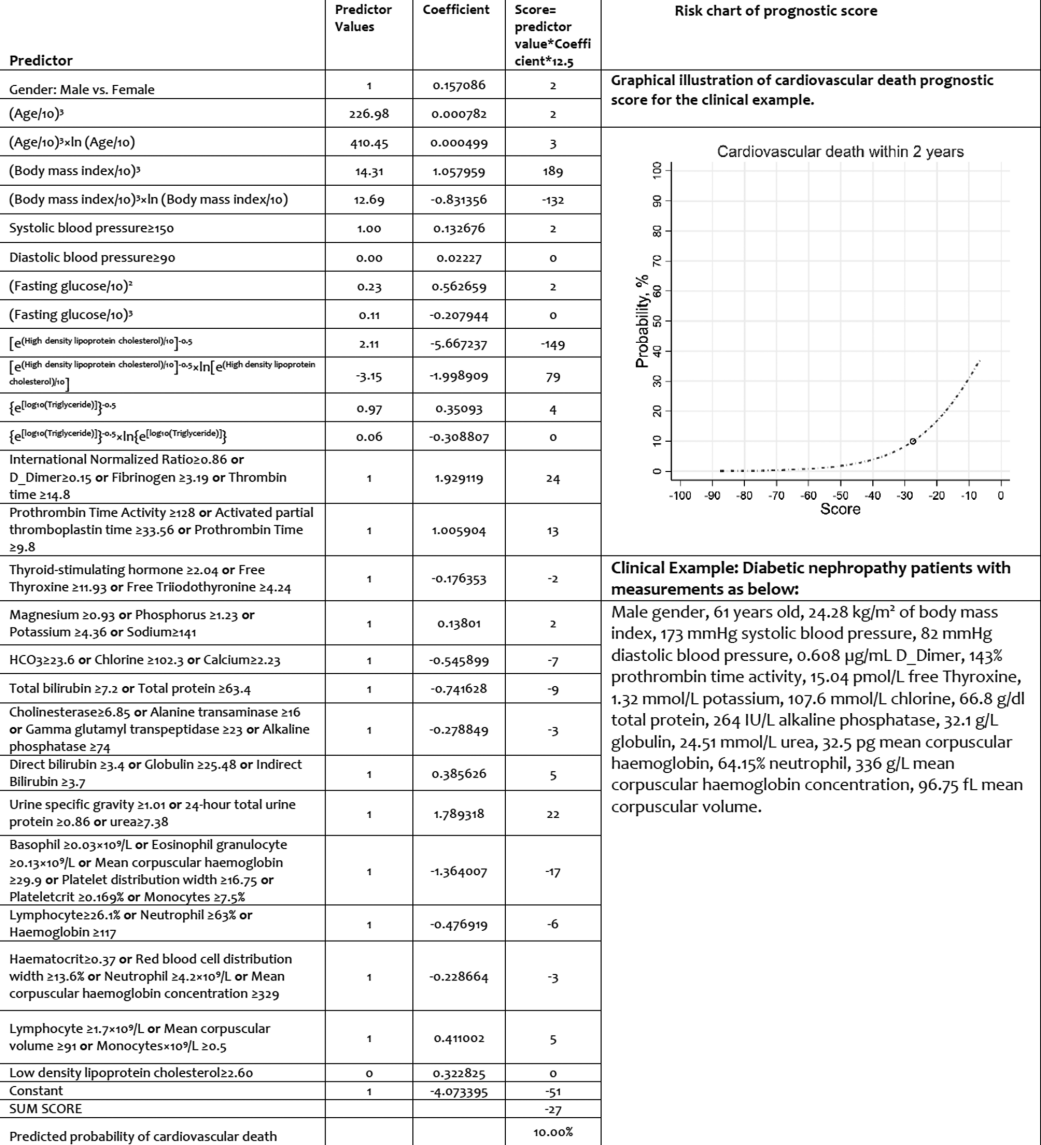
Practical prognostic score charts for predicting cardiovascular death among patients with incident DN. DN; diabetic nephropathy.

Figure 3 gives a clinical example of the application of prognostic score charts with graphical illustrations for the cardiovascular death prediction model to predict 2-year risk of cardiovascular deaths.

## Discussion

### Main findings

A new risk prediction algorithm has been developed in this study to quantify the absolute risk of cardiovascular death within 2 years in a prospective cohort of Chinese patients with incident diagnosis of DN. The prediction model was then externally validated in another independent prospective cohort. The risk prediction model demonstrated useful discrimination and excellent calibration, with C-statistics of >0.70 both in the derivation cohort and external validation cohort. The risk prediction model was derived from clinical measurements routinely recorded and accessible in diabetes care settings (outpatients and inpatients), indicating that these can be readily applied in routine diabetes care (eg, by embedding in medical administrable software).

### Comparison with previous studies

van der Sande *et al* developed a prediction model to predict cardiovascular events within 3 years among patients with prevalent DN treated with angiotensin receptor blockers.^18^ Age, gender, smoking, SBP, urinary albumin/creatinine ratio, eGFR, albumin and phosphate were included as predictors. However, the model performed poorly and yielded a C-statistic of 0.61 (95% CI 0.59 to 0.64) with a general slope calibration >1.00 (ie, the model overpredicted risk).

Previous risk prediction models have not fully addressed cardiovascular disease itself as the primary reason for death in patients with newly diagnosed DN. Being aware of the absolute risk of cardiovascular death in the following 2 years could help clinicians in their discussions with patients, and the urgency and intensity with which they provide cardiovascular event preventative care to patients with a high-risk profile, and could lead to a reduction in health cost overall. Implementation could be tested using a randomized controlled trial, with health economic assessment, and could include embedding alerts into practice software and increasing patient awareness of their risk.

### Comparison with other risk score

The UKPDS risk score has been developed for patients who have newly diagnosed type 2 diabetes to estimate their 1–10 years CVD risk. The algorithm performs well for the general population with type 2 diabetes.^16^ We applied the UKPDS risk score in the external validation cohort and found it substantially underestimated the 2-year CVD risk predicted. This might be due to levels of the UKPDS predictors (age, sex, glucose, SBP and lipid levels) being different in the population with DN compared with the wider type 2 diabetes population.^3^ This suggests that using the UKPDS risk engine to guide clinical management might not be suitable among patients with type 2 diabetes and DN. Our new risk score would be a more specific risk calculator for such patients.

### Strengths and limitations

There are several advantages in our prediction model over those applied elsewhere. The risk score is on the basis of absolute risk derivation and validation in two prospective cohorts. Demographic and clinical measurements routinely recorded both in outpatient and inpatient settings were used to derive the prediction model. This indicates that it can be readily embedded into online tools for their application in outpatient or inpatient settings. Furthermore, compared with patients with type 2 diabetes, those with DN are on a fast-track to progress to a cardiovascular event, including premature cardiovascular death.^1 3^ The identification of individuals at high risk of cardiovascular deaths in the short term could help clinicians to prioritize new therapy (such as Sodium Glucose Co-Transporter-2 (SGLT-2) inhibitors) to delay a fatal (or non-fatal) cardiovascular event.

The approaches used to develop and validate the present model are similar to those for other risk prediction models derived from the Clinical Practice Research Datalink (CPRD) and QResearch studies.^19 20^ The predictors in our final model are accurate and reliable clinical variables routinely recorded in outpatient and inpatient settings and updated and reviewed for patients with DN having type 2 diabetes and are less varied than in other datasets. Moreover, the volume of missing values was relatively low, which would be less likely to lead to variation in potential external applications, although we applied multiple imputation. Caution is needed in interpreting the association between these predictors and the outcome, as the multiple imputation used might introduce information bias as the proportion of missing data was high with some predictors. This is likely to be less important as the aim of this study was to develop a risk score rather than investigating the causal association between exposure (predictor) and the outcome: multiple imputation of predictors is a good approach for model derivation to improve prediction accuracy.^21^

In this study, the definition of an incident clinical diagnosis of DN was based on existing medical records. Naturally, the timing of the actual onset of DN would be unknown (as with many other non-communicable diseases), and the inclusion of prevalent cases would therefore be possible. However, renal function tests (ie, creatinine) were used routinely among patients with type 2 diabetes to prospectively screen for chronic kidney disease and the possibility of enrolling prevalent cases was low. Restricted by current sample size and to facilitate utilization of the risk score, only polynomial terms of traditionally well-known prognostic factors (age, BMI, SBP, DBP, glucose, HbA1c, total cholesterol, LDL, HDL and triglycerides) were tested; polynomial terms of each laboratory items were not tested in the current study. Further studies with large sample size and more polynomial terms of laboratory items are warranted. We acknowledge that antidiabetes treatments, diabetes duration, history of cardiovascular disease, antihypertensive treatment, lifestyle risk factors (like smoking) and other comorbidities were not taken into account as a result of limitations in the original data. However, some of these prognostic factors are very common in people with diabetes (such as antihypertensive treatment which is used in 81.2% of patients with type 2 diabetes^22^), and as a result, would be less discriminatory in the model. We also believe that at least some of the clinical measurements incorporated in the prediction model could serve as proxies for these inaccessible predictors. Due to the sample size of the derivation and validation cohorts, further independent external validations (eg, external data from other low-income and middle-income countries) with large sample size are warranted. As the risk score was derived and externally validated in Chinese population with type 2 diabetes and incident DN, further validations in other ethnic groups are warranted. Multicollinearity could exist between predictors in this study. However, instead to quantifying a causal association, the goal of this study was to make a prediction that would be less likely to be influenced by multicollinearity.^23^

## Conclusions

In conclusion, this is the first study to derive a prediction model to quantify the 2-year absolute risk of cardiovascular death among patients with type 2 diabetes and newly diagnosed DN. Our risk algorithm has three useful implications for DN practice. First, the risk score can be used as a screening tool to identify patients with high probability of cardiovascular death. The risk score is based on readily accessible clinical information routinely recorded either in outpatient setting or inpatient setting and evaluated by diabetes management teams. It can be readily embedded into heath administration computer systems or developed into a mobile application for a handheld device for ease of use. Second, the risk prediction score could be applied to establish new treatment thresholds in diabetes clinical practice through consensus development of guidelines. Third, this new risk score is a more specific risk calculator for DN compared with the UKPDS risk score.
