# Supplementary material for: Derivation and external validation of a risk prediction algorithm to estimate future risk of cardiovascular death among patients with type 2 diabetes and incident diabetic nephropathy: prospective cohort study
Source: BMJ Open Diabetes Res Care. 2019 Nov 13;7(1):e000735. doi: 10.1136/bmjdrc-2019-000735 (PMC6861120; doi:10.1136/bmjdrc-2019-000735)
Supplement: Supplementary data [file bmjdrc-2019-000735supp001.pdf]

Supplemental Table 1. Potential predictors to derive the prediction score

NO	Predictors	Terms included before backward elimination	Included in the final model
1	Gender	Binary (Male=1; Female=0)	Yes
2	Age	2 polynomial terms (as presented in Table-2)	Yes
3	Body mass index	2 polynomial terms (as presented in Table-2)	Yes
4	Systolic blood pressure	Binary (=1 if SBP≥150mmHg, =0 if SBP<150mmHg	Yes
5	Diastolic blood pressure	Binary (=1 if DBP≥90mmHg, =0 if SBP<90mmHg	Yes
6	Fasting glucose	2 polynomial terms (as presented in Table-2)	Yes
7	**HbA1c**	**Binary (=1 if HbA1c≥62.8mmol/mol, =0 if HbA1c<62.8mmol/mol)**	**No**
8	**Total cholesterol**	**Binary ((=1 if LDL≥4.3mmol/L, =0 if LDL<4.3mmol/L)**	**No**
9	High density lipoprotein	2 polynomial terms (as presented in Table-2)	Yes
10	Triglyceride	2 polynomial terms (as presented in Table-2)	Yes
11	Low density lipoprotein	Binary ((=1 if LDL≥2.6mmol/L, =0 if LDL<2.6mmol/L)	Yes
12	International Normalized Ratio≥0.86 or D_Dimer≥0.15 or Fibrinogen ≥3.19 or Thrombin time ≥14.8	Binary (=1 if any item presented in the left cell above the threshold, =0 if all items presented in the left cell below the threshold)	Yes
13	Prothrombin Time Activity ≥128 or Activated partial thromboplastin time ≥33.56 or Prothrombin Time ≥9.8	Binary (=1 if any item presented in the left cell above the threshold, =0 if all items presented in the left cell below the threshold)	Yes
14	Thyroid-stimulating hormone ≥2.04 or Free Thyroxine ≥11.93 or Free Triiodothyronine ≥4.24	Binary (=1 if any item presented in the left cell above the threshold, =0 if all items presented in the left cell below the threshold)	Yes
15	Magnesium ≥0.93 or Phosphorus ≥1.23 or Potassium ≥4.36 or Sodium≥141	Binary (=1 if any item presented in the left cell above the threshold, =0 if all items presented in the left cell below the threshold)	Yes
16	HCO3≥23.6 or Chlorine ≥102.3 or Calcium≥2.23	Binary (=1 if any item presented in the left cell above the threshold, =0 if all items presented in the left cell below the threshold)	Yes
17	Total bilirubin ≥7.2 or Total protein ≥63.4	Binary (=1 if any item presented in the left cell above the threshold, =0 if all items presented in the left cell below the threshold)	Yes
18	Cholinesterase≥6.85 or Alanine transaminase ≥16 or Gamma glutamyl transtransferase ≥23 or Alkaline phosphatase ≥74	Binary (=1 if any item presented in the left cell above the threshold, =0 if all items presented in the left cell below the threshold)	Yes
19	Direct bilirubin ≥3.4 or Globulin ≥25.48 or Indirect Bilirubin ≥3.7	Binary (=1 if any item presented in the left cell above the threshold, =0 if all items presented in the left cell below the threshold)	Yes
20	Prealbumin≥234.04 mg/dl or Total bile acid≥3.90μmol/L or Albumin≥39.60 g/L or Aspartate transaminase ≥ 17.00 U/L	Binary (=1 if any item presented in the left cell above the threshold, =0 if all items presented in the left cell below the threshold)	No
21	Urine specific gravity ≥1.01 or 24-hour total urine protein ≥0.86 or urea≥7.38	Binary (=1 if any item presented in the left cell above the threshold, =0 if all items presented in the left cell below the threshold)	Yes
22	24-hour total urine amount≥2.0L or Urine microalbumin≥149.07mg/L/24-hour or pH of urine sample ≥6.0 or Urine total protein≥421.10mg/24 h or Creatinine ≥ 86.0μmol/L or Urine acid≥303.0μmol/L or Estimated glomerular filtration rate≥35.79 mL/min/1.73m2	Binary (=1 if any item presented in the left cell above the threshold, =0 if all items presented in the left cell below the threshold)	No
23	Basophil ≥0.03×10⁹/L or Eosinophil granulocyte ≥0.13×10⁹/L or Mean corpuscular haemoglobin ≥29.9 or Platelet distribution width ≥16.75 or Plateletcrit ≥0.169% or Monocytes ≥7.5%	Binary (=1 if any item presented in the left cell above the threshold, =0 if all items presented in the left cell below the threshold)	Yes
24	Lymphocyte≥26.1% or Neutrophil ≥63% or Haemoglobin ≥117	Binary (=1 if any item presented in the left cell above the threshold, =0 if all items presented in the left cell below the threshold)	Yes

25	Haematocrit≥0.37 or Red blood cell distribution width ≥13.6% or Neutrophil ≥4.2×10⁹/L or Mean corpuscular haemoglobin concentration ≥329 g/L	Binary (=1 if any item presented in the left cell above the threshold, =0 if all items presented in the left cell below the threshold)	Yes
26	Lymphocyte ≥1.7×10⁹/L or Mean corpuscular volume ≥91 fL or Monocytes×10⁹/L ≥0.5	Binary (=1 if any item presented in the left cell above the threshold, =0 if all items presented in the left cell below the threshold)	Yes
27	Basophil≥0.5% or Eosinophil granulocyte≥2.0% or Mean platelet volume≥91.0fL or Plateletcrit≥0.17% or Red blood cell≥4.29×10¹²/L or White blood cell count≥6.55×10⁹/L	Binary (=1 if any item presented in the left cell above the threshold, =0 if all items presented in the left cell below the threshold)	No

Cut-offs for the continuous laboratory test items were chosen by testing the 25th, 50th and 75th percentile in the derivation cohort was tested. The model with predictors using median as cut-offs was the best fitted model.
